# Cost-Effectiveness of Two Decision Strategies for Shunt Use During Carotid Endarterectomy

**DOI:** 10.1007/s00268-017-4085-5

**Published:** 2017-06-16

**Authors:** Joe L. P. Kolkert, Rolf H. H. Groenwold, Vanessa J. Leijdekkers, Joep ter Haar, Clark J. Zeebregts, Anco Vahl

**Affiliations:** 1 Department of Surgery, Onze Lieve Vrouwe Gasthuis, P.O. Box 95500, 1090 HM Amsterdam, The Netherlands; 2 Department of Surgery, Division of Vascular and Transplant Surgery, Radboudumc, Geert Grooteplein-Zuid 10, P.O. Box 9101, 6500 HB Nijmegen, The Netherlands; 3 Julius Center for Health Sciences and Primary Care, University Medical Center Utrecht, P.O. Box 85500, 3508 GA Utrecht, The Netherlands; 4 Department of Surgery, Sint Lucas Andreas Ziekenhuis, P.O. Box 9243, 1006 AE Amsterdam, The Netherlands; 5 Department of Surgery (Division of Vascular Surgery), University Medical Center Groningen, University of Groningen, P.O. Box 30001, 9700 RB Groningen, The Netherlands

## Abstract

**Background:**

Arterial shunting during carotid endarterectomy (CEA) is essential in some patients because of insufficient cerebral perfusion during cross-clamping. However, the optimal diagnostic modality identifying these patients is still debated. None of the currently used modalities has been proved superior to another. The aim of this study was to assess the cost-effectiveness of two modalities, stump pressure measurement (SPM) versus electroencephalography (EEG) combined with transcranial Doppler (TCD) during CEA.

**Methods:**

Two retrospective cohorts of consecutive patients undergoing CEA with different intraoperative neuromonitoring strategies (SPM vs. EEG/TCD) were analyzed. Clinical data were collected from patient hospital records. Primary clinical outcome was in-hospital stroke or death. Total admission costs were calculated based on volumes of healthcare resources. Analyses of effects and costs were adjusted for clinical differences between patients by means of a propensity score, and cost-effectiveness was estimated.

**Results:**

A total of 503 (239 SPM; 264 EEG/TCD) patients were included, of whom 19 sustained a stroke or died during admission (3.3 vs. 4.2%, respectively, adjusted risk difference 1.3% (95% CI −2.3–4.8%)). Median total costs were €4946 (IQR 4424–6173) in the SPM group versus €7447 (IQR 6890–8675) in the EEG/TCD group. Costs for neurophysiologic assessments were the main determinant for the difference.

**Conclusions:**

Given the evidence provided by this small retrospective study, SPM would be the favored strategy for intraoperative neuromonitoring if cost-effectiveness was taken into account when deciding which strategy to adopt.

## Introduction

Carotid endarterectomy (CEA) is a prophylactic intervention to prevent future ischemic events in patients with a symptomatic carotid stenosis. However, patients involved are exposed to a perioperative stroke or death risk of approximately 3%, of which one-third occurs intraoperatively due to embolization or cerebral ischemia during cross-clamping [1–4]. The use of a shunt might reduce cerebral ischemia by maintaining ipsilateral flow but is still debated since it has only been shown necessary in 10–14% of patients undergoing CEA under local or regional anesthesia, which can be considered as reference standard. [5–7]. Moreover, shunting itself is associated with complications too, including atheromatous or air emboli, arterial dissection, and acute arterial occlusion [8–11]. Therefore, many surgeons advocate selective shunting, instead of routine shunting, in those patients at high risk of cerebral ischemia. Methods frequently used to evaluate cerebral perfusion during cross-clamping and therewith the need for selective shunting include computerized electroencephalography (EEG), transcranial Doppler (TCD), stump pressure measurement (SPM), and neurologic examination when CEA is performed under regional or local anesthesia. None of these methods has been proved to be superior with regard to intraoperative stroke risk reduction [9]. These methods do, however, differ in labor intensity and might consequently be associated with different costs and/or cost-effectiveness.

Considering the increasing costs of healthcare and decreasing health resources, costs might be taken into account when deciding which strategy to adopt. The primary aim of this study was to assess the cost-effectiveness of two modalities used to assess cerebral perfusion after cross-clamping: SPM versus combined EEG and TCD.

## Methods

### Study design and patients

**Fig. 1:**
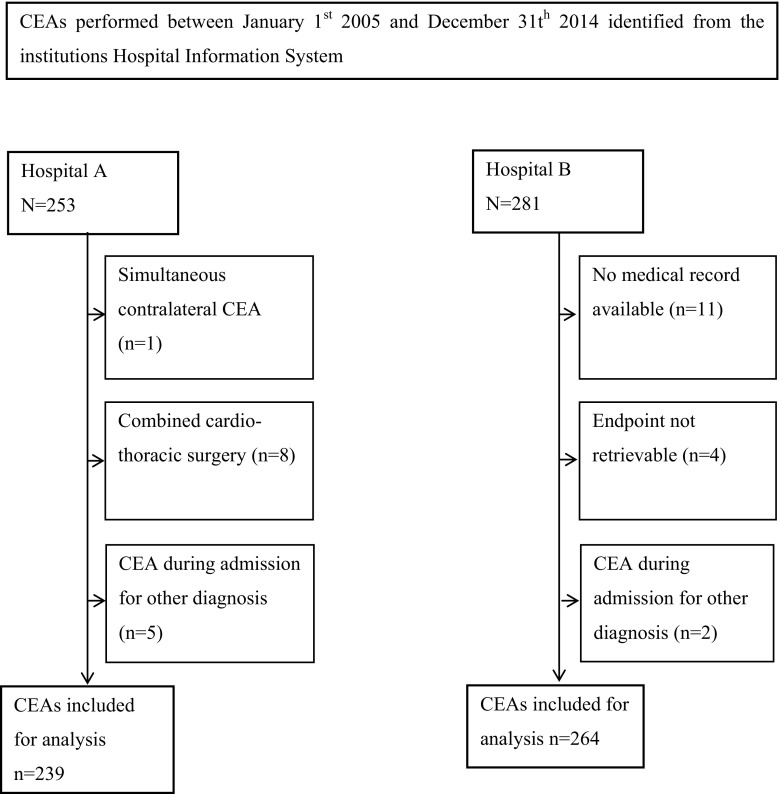
CEAs performed between January 1, 2005, and December 31, 2014, identified from the institutions hospital information system

All consecutive patients who underwent CEA between January 2005 and December 2014 in two midsize teaching hospitals (Onze Lieve Vrouwe Gasthuis Amsterdam, hospital A; Sint Lucas Andreas Ziekenhuis Amsterdam, hospital B; the Netherlands) were included in this study. Patients undergoing synchronous coronary artery bypass grafting, patients initially admitted for another reason but whom underwent a CEA during the admission because of an in-hospital TIA or stroke, and those for whom medical records were not available or costs could not be extracted from the hospital information systems were excluded, leaving 503 evaluable admissions for CEA (Fig. 1).

Demographics, comorbidities, cardiovascular risk factors, procedural details, and postoperative outcome were collected from patient hospital records (operation reports, radiology reports, and correspondence). Volumes of healthcare resources from the index admission were extracted from the hospital information systems.

Retrospective patients’ files research is not under the scope of the Dutch ACT on Medical Scientific Research involving Human Beings (WMO). The institutional review board approved the protocol, data collection, and study design (WO 15.007), and therefore, patient informed consent was not required. Patient data were analyzed anonymously.

### Shunting strategy

Except for shunting strategy, the procedure of CEA was similar in both hospitals including arteriotomy with patch closure. In hospital A, SPM was used to determine the need for a shunt, and in hospital B, EEG combined with TCD. SPM was performed using a 21-gauge needle connected to a pressure transducer by a fluid-filled pressurized tubing. Prior to cross-clamping, systolic blood pressure was brought to baseline (preoperative) values and the pressurized system was zero-referenced against ambient atmospheric pressure, keeping the needle at level of the common carotid artery. After cross-clamping the external and common carotid arteries, the needle was inserted into the common carotid artery distally to the clamp. A systolic stump pressure lower than 50 mmHg was considered indicative for shunt insertion. The procedures for EEG monitoring and TCD assessment in hospital B have been described in detail elsewhere [12, 13]. Standard neurophysiologic assessments consisted of EEG monitoring and TCD assessment prior to as well as during CEA, and TCD postoperatively.

### Cost analysis

This analysis was performed from a provider perspective taking into account true costs made during the index hospital admission, defined as the admission for scheduled CEA. For cost analysis, all costs associated with the index admission were considered using standardized methods [14]. Costs made before admission or after discharge from hospital were not taken into account. All unit costs were derived and calculated from the 2013 financial ledger of hospital A, using activity-based costing to accurately measure operation costs for CEA and hospital day unit costs (intensive care unit costs and general ward costs were defined separately). Hospital day unit costs included the costs for physician care, nursing, materials, medication, writing-off equipment, housing, and other overhead costs. Operation costs include specialist’ fee, costs of personnel, equipment, materials, and overhead costs. Additional costs for the department of clinical neurophysiology for performing perioperative EEG and TCD were calculated separately for those patients treated in hospital B and also include specialist ‘fee’ cost for personnel equipment, materials, and overhead costs. Volumes of blood products, radiology, laboratory tests, physiotherapy, consultation of other specialties, etc., were extracted from the hospital information systems. Total costs were calculated as the summed product of volumes and resources used and their corresponding unit costs. Because costs between the two hospitals might differ due to contracts with different suppliers of materials and equipment, and the fact that healthcare reimbursements in the Netherlands are based upon agreements between individual hospitals and insurance companies, costs were calculated as if all patients had been treated in the same hospital (hospital A).

Primary outcome measures for the cost-effectiveness analyses were in-hospital stroke or death, which was obtained from medical records and all costs associated with the admission. This primary end point was chosen since stroke and death were assumed to influence hospital costs due to longer hospital stay, including ICU admission, and costs for instance additional imaging and specialist’ consultation. Secondary outcome measures were hospital stay, duration of operation (total time between entering and leaving the operation room), shunt use, and complication rate.

### Statistical analysis

Descriptive statistics (mean, median, proportion) of patient characteristics were determined stratified by hospital. Adjustment for difference in patient characteristics between both strategies, i.e., confounding, was done by propensity score analysis [15]. First, a logistic regression model was fit, regressing the strategy on multiple confounders, i.e., patients’ age, sex, blood pressure (hypertension vs. normal tension), coronary artery disease, PAD, diabetes mellitus, history of smoking, indication for the CEA, and degree of ipsilateral and contralateral stenosis. Subsequently, the estimated propensity scores, summarizing the information of multiple confounders, were included as a single covariate in the models estimating the differences in outcome incidence and costs between the two strategies. The effect of strategy on the incidence of outcome was quantified by means of a risk difference. Therefore, both effects and costs were estimated by means of a linear regression model, resulting in estimates of the risk difference in outcome and the difference in costs between the two hospitals, each with corresponding 95% confidence intervals. Then, the joint cost-effectiveness was estimated by plotting 1000 bootstrap estimates of costs and effects in a cost-effectiveness plane [16].

A sensitivity analysis was performed to assess the potential impact of misclassification on the outcome, specifically to assess the impact of an event going undetected in one of the two hospitals. For that, a random subject who did not experience the outcome was assumed to have the outcome and all analyses were repeated. This procedure was repeated 1000 times, and estimates of costs and effects were plotted in a cost-effectiveness plane. Calculations were done separately for the SPM and the EEG/TCD groups. All analyses were performed in R for Windows, version 3.0.3 (R Development Core Team, 2008).

## Results

### Patients

A total of 503 admissions for CEA were included in this study (Fig. 1). Seventy-one percent was male. The mean age was 69.5 ± 9.9 years. The vast majority of patients (97.4%) had symptomatic carotid disease. SPM was used in 239 CEAs, EEG/TCD in 264. The technical success rate was 98.3% for SPM and 93.2% for EEG/TCD (in 6.8% only EEG recordings were used due to an absent temporal window). The EEG/TCD group was slightly older and consisted of more female patients. Moreover, less concomitant peripheral vascular disease has been recorded in this group. Patient demographics and indication for treatment are shown in Table 1.

**Table 1:** Baseline characteristics of both cohorts

Variable	SPM	EEG/TCD	*P* value
	*n* = 239 No (%)	*n* = 264 No (%)	
Age, mean ± SD (years)	68.6 ± 9.2	70.3 ± 10. 5	0.045
Sex, male	181 (75.7)	178 (67.4)	0.050
Risk factors			
Hypertension	144 (60.3)	166 (62.9)	0.608
CAD	66 (27.6)	71 (26.9)	0.935
PAD	55 (23.0)	35 (13.3)	0.006
Diabetes mellitus	62 (25.9)	76 (28.8)	0.539
History of smoking	184 (77.0)	187 (70.8)	0.143
Index event			
Asymptomatic	8 (3.3)	5 (1.9)	0.654
Amaurosis fugax	56 (23.4)	57 (21.6)	
TIA	78 (32.6)	95 (36.0)	
Stroke	97 (40.6)	107 (40.5)	
Time index event to CEA^#^			
0–3 days	3 (1.4)	4 (1.8)	0.227^a^
4–7 days	27 (12.8)	17 (7.6)	
8–14 days	73 (34.8)	73 (32.4)	
>14 days	107 (51.0)	131 (58.2)	
Degree of ispilateral stenosis			
50–99%	29 (12.1)	37 (14.0)	0.623
70–99%	210 (87.9)	227 (86.0)	
Degree of contralateral stenosis^$^			
0–69%	182 (76.2)	210 (79.5)	0.287
70–99%	29 (12.1)	34 (12.9)	
Occlusion	28 (11.7)	20 (7.6)	

*SPM* stump pressure measurement, *EEG* electroencephalography, *TCD* transcranial Doppler, *SD* standard deviation, *CAD* coronary artery disease, *PAD* peripheral artery disease, *TIA* transient ischemic attack

^#^Exact date of event was retrievable for 210 patients in the SPM group and 225 patients in the EEG/TCD group

^$^The granularity of recording of contralateral stenosis did not allow for further categorization of stenosis <70%

^a^Based on likelihood ratio test

### Clinical outcome

The mean overall operation time did not differ between the two strategies (SPM 145 ± 34 min; EEG/TCD 148 ± 31 min; *P* = 0.36). SPM indicated shunt use in 113 patients (47.3%) and EEG/TCD in 28 patients (10.6%). Contralateral occlusion was associated with a higher shunt rate (69 vs. 23%, relative risk 3.0; 95% CI 2.3–3.9). Median hospital stay was 4 days (IQR 3–6) after SPM versus 3 days (IQR 3–5) after EEG/TCD (Table 2).

**Table 2:** Operative and clinical outcome

Variable	SPM	EEG/TCD	*P* value
	*n* = 239	*n* = 264	
Stump pressure, mean ± SD (mmHg)	48.2 ± 19.6	–	
Use of shunt, no (%)	113 (47.3)	28 (10.6)	<0.001
Operation time,^a^ mean ± SD (min)	145 ± 34	148 ± 31	0.36
Hospital stay, median (IQR) (days)	4 (3-6)	3 (3-5)	<0.001
Complication, no (%)			
None	200 (83.7)	226 (85.6)	<0.001^b^
Bleeding	9 (3.8)	23 (8.7)	
Myocardial infarction	3 (1.3)	0	
Non-fatal stroke	5 (2.1)	10 (3.8)	
Death	3 (1.3)	1 (0.4)	
Nerve injury	13 (5.4)	2 (0.8)	
Other	6 (2.5)	2 (0.8)	
Stroke/death, no (%)	8 (3.3)	11 (4.2)	0.63
Re-intervention no (%)	14 (5.9)	12 (4.5)	0.51
Total costs (€), median (IQR)	4946 (4424–6173)	7447 (6890–8675)	<0.001

*SPM* stump pressure measurement, *EEG* electroencephalography, *TCD* transcranial Doppler *SD* standard deviation, *IQR* interquartile range

^a^Time interval between entering and leaving the operation room

^b^Based on likelihood ratio test

In-hospital stroke or death rate did not differ between the two strategies (SPM 3.3% vs. EEG/TCD 4.2%; adjusted risk difference 1.3% (95% CI −2.3–4.8%)). There were four postoperative deaths of which two following a stroke. Of all 17 perioperative strokes, five had an intraoperative onset (SPM two; EEG/TCD three), eight occurred postoperatively (four in both groups), and four were associated with hyperperfusion syndrome (SPM one; EEG/TCD three). A shunt had been used in three (SPM one; EEG/TCD two) out of five patients suffering an intraoperative stroke.

### Costs, cost-effectiveness, and uncertainty assessment

Median total costs of hospitalization for CEA were €4946 (IQR 4424–6173) in the SPM group versus €7447 (IQR 6890–8675) in the EEG/TCD group (*P* < 0.001). The adjusted difference in costs was €2053 (95% CI 1424–2682). Main determinant for this difference was the costs for neurophysiologic assessments (mean €2012 per patient). In both groups, there were no differences in hospital costs between patients in whom a shunt had been inserted and those in whom had not: SPM €4864 (IQR 4476–5836) versus €4979 (IQR 4727–6030), *P* = 0.192; EEG/TCD €7445 (IQR 6890–8680) versus €7523 (IQR 6923–9064), *P* = 0.638. Table 3 shows the mean resource use and corresponding costs in both cohorts.

**Table 3:** Volumes and costs per patient of healthcare resources

Cost item	Unit costs (€)	Mean resource use per patient		Mean costs per patient (€)	
		SPM	EEG/TCD	SPM	EEG/TCD
Hospitalization (per day)					
General ward	437.97	5.400	4.900	2365.04	2146.05
ICU	1110.53	0.060	0.261	66.63	290.25
Surgery (mean)					
Primary operation	2969.00	1.000	1.000	2969.00	2933.80^$^
Reoperation	1855.22	0.059	0.045	108.60	84.33
*Diagnostics*					
Neurophysiologic assessment					
EEG	380.65		1.041		391.26
TCD	177.51		2.250		399.40
Complete intraoperative monitoring (EEG + TCD)	1217.00		0.932		1134.24
Radiology					
X-ray thorax	28.55	0.295	0.186	8.42	5.30
Ultrasound neck	80.73	0.047	0.144	3.63	11.62
CT brain	111.80	0.053	0.136	5.93	15.25
MRI brain	390.75	0.003	0.024	1.17	9.26
Other diagnostic modality		0.076	0.109	14.05	7.83
Laboratory tests					
HCC		25.397	25.977	43.99	54.37
Microbiology		0.870	0.0678	17.95	13.01
Consultation^#^					
Physiotherapy	57.39	0.071	1.078	4.08	61.84
Occupational therapy	28.69	0.833	0.159	23.81	4.56
Speech therapy	69.29	0.326	0.144	22.61	7.87
Other costs				283.93	499.01
Total				5938.90	7994.98
(total median costs per patient)				(4946.00)	(7447)

*SPM* stump pressure measurement, *EEG* electroencephalography, *TCD* transcranial Doppler, *ICU* intensive care unit, *CT* computed tomography, *MRI* magnetic resonance imaging, *HCC* hematology and clinical chemistry

^#^ Per consult

^$^ Average shunt use included in price

^¶^ Both EEG and TCD registered in 93% of all patients

**Fig. 2:**
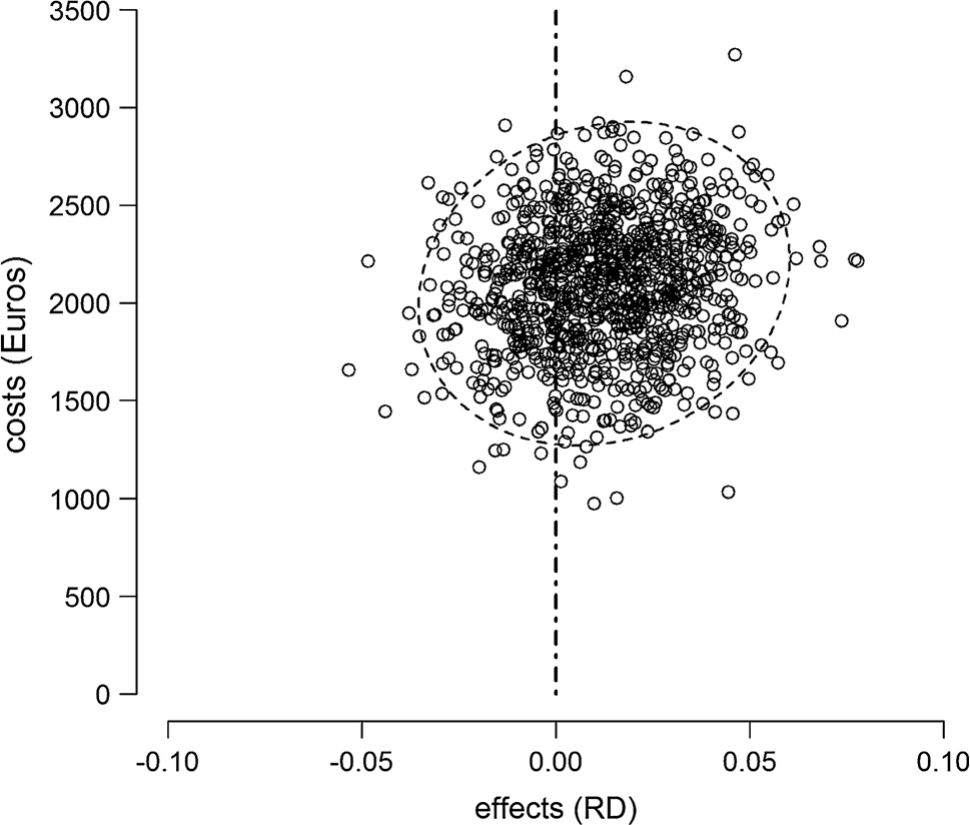
Cost-effectiveness of SPM versus EEG/TCD. The effect (stroke/death) is expressed as risk difference (RD). Estimates are adjusted for age, risk factors (smoking, coronary artery disease, peripheral artery disease, diabetes mellitus), index event, and ipsilateral and contralateral degree of stenosis

Figure 2 shows the ninety-five percent confidence ellipse of the cost difference between the different strategies. The bootstrapping results are almost divided equally between both upper quadrants of the figure, indicating no significant difference in stroke/death rates, but higher costs in the EEG/TCD group.

**Fig. 3:**
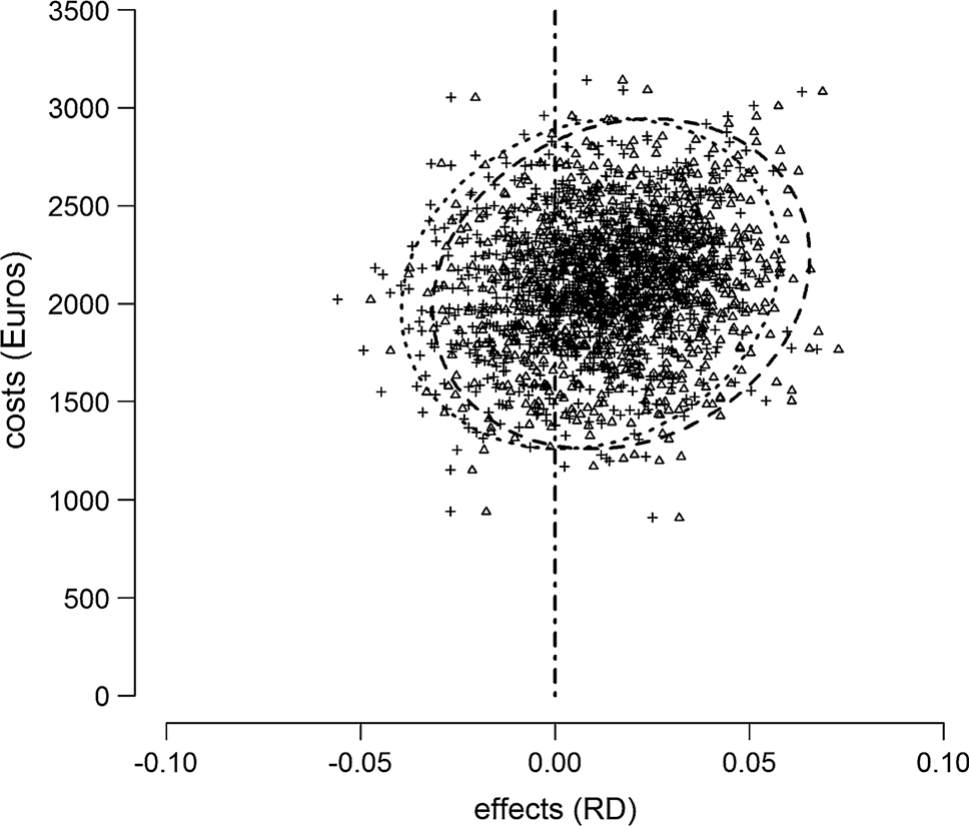
Sensitivity analysis to assess the potential impact of misclassification on the outcome, specifically to assess the impact of an event going undetected. *Triangles* (and *dashed confidence ellipse*) represent a scenario in which a random non-event in hospital A is converted into an event; the *crosses* (*dotted confidence ellipse*) represent a scenario in which a random non-event in hospital B is converted into an event

A sensitivity analysis showed that misclassification of an event in one of the both groups would not materially impact the results of the cost-effectiveness analysis as shown in Fig. 2 (Fig. 3).

## Discussion

This study shows that SPM was less costly without a significant difference in adverse events in terms of stroke or death rate compared to EEG/TCD. The mean adjusted difference in total admission costs for CEA was €2053 and largely attributable to the perioperative neurophysiologic assessments when using EEG/TCD. Perioperative morbidity and mortality in our cohorts were comparable to those found in other CEA studies. We consider our cost-effectiveness analysis of interest. The different modalities used in the participating hospitals have been chosen for reasons in the past. SPM is less frequently used nowadays since it does not provide any information on cerebral perfusion following cross-clamping and neither accounts for the occurrence of cerebral ischemia during plaque removal and arterial reconstruction. EEG/TCD does, on the other hand, provide this information, but no benefit in terms of perioperative stroke risk reduction can be found in the literature. EEG/TCD is, however, associated with higher costs.

A reason to adopt a selective shunting strategy instead of routine shunting can be the fact that shunts are not necessarily benign and have been associated with complications [17–20]. The shunt rate in the SPM group was quite high, probably due to the chosen systolic cutoff point of 50 mmHg as indicative for shunt insertion. Yet, the optimal stump pressure cutoff point is still controversial. Several studies attempted to determine an optimal stump pressure threshold in patients undergoing CEA under regional anesthesia and found increasing accuracy by lowering the threshold from 50 to 40 mmHg. Unnecessary shunt use decreased by 20–25%, and the erroneously non-shunted rate was kept between one and three percent [7, 8, 21]. The threshold for shunt insertion in hospital A may therefore have resulted in the unnecessary high shunt rate. Since three out of five intraoperative strokes in this study occurred in shunted patients, the threshold for shunt insertion was lowered to 40 mmHg after the study. It is, however, not certain that the intraoperative stroke in those three shunted patients was due to the shunt placement itself. The actual need for a shunt might indicate that these patients were prone for ischemia anyway Moreover, in search for the ideal stump pressure, a certain number of false-positive outcomes (unnecessary use of a shunt) have to be accepted in order to keep the false negative (erroneously not-shunted patients) as low as possible.

On the contrary, the shunt rate found in our EEG/TCD group approaches those found in studies where CEA was performed in awake patients, which is considered as reference standard [5, 7, 21]. EEG and TCD allow for continuous monitoring and can therefore, in contrast to SPM, also detect a malfunctioning shunt. Moreover, TCD provides information about the occurrence of microemboli, allowing adaptation of surgical technique and handling and might also be useful in the early postoperative phase to predict cerebral hyperperfusion syndrome (CHS) and upcoming thrombotic stroke [22–24]. This shunt rate is, however, not necessarily worth pursuing, since low false-positive rates usually come at the expense of the sensitivity. Only few studies have determined the accuracy of EEG recordings in awake patients (reference standard). These studies show positive predictive values (PPVs) of EEG detecting true neurologic deterioration ranging from 40.9 to 90.0%. Thus, if these patients had undergone surgery under general anesthesia, a shunt would have been placed unnecessarily in 10.0–59.1% of the patients. Negative predictive values were found ranging from 94.4 to 99.2%, meaning that in 0.8–5.6% of the patients, EEG would have failed to detect neurologic deficit and a shunt would have been wrongly withheld [7, 25–27]. PPVs and NPVs of TCD as sole modality in detecting cerebral ischemia range from 19 to 75% and 97 to 99%, respectively, depending on criteria used as indicative for shunting [28–30]. These varying figures might reflect differences in subjective interpretations of EEG tracings and/or TCD recordings. Both techniques therefore require well-trained personnel, are time-consuming, and thus are costly.

The mean hospital costs for CEA found in our series are comparable to those found in other studies. Recently, Buisman et al. [31] determined hospital resource use and costs for ischemic stroke and TIA in the Netherlands. Costs were estimated at € 6836 ± 2862, with surgery and hospitalization (average 4.8 inpatient days) as main determinants, accounting for 51 and 34% of the total costs, respectively, similar to our series.

It is important to note that a variety of methods other than those we studied are available to determine the need for a shunt. Moreover, some surgeons prefer the routine use of a shunt, and others perform CEA under loco-regional anesthesia making neuromonitoring unnecessary. In the Netherlands, a selective shunting strategy is used in the large majority of CEAs (>90%). EEG is most frequently used (43%), followed by EEG combined with TCD (40%). SPM is only used in 1.6% of all CEAs [4]. A recent meta-analysis could not demonstrate a clinical benefit of one strategy above the other in terms of 30-day death or stroke rate [9]. Both routine shunting as well as selective shunting, whatever modality used, seem to be acceptable.

There are several limitations to our study. First, due to its retrospective character, this study had to rely on completeness of existing data registries. Several patients were excluded due to missing data (Fig. 1). Furthermore, cost analysis is dependent on the accuracy of registration of resources used during the admission. Under-registration can lead to erroneous lower costs, but this may have occurred in both groups. Moreover, since there was a rather large difference in costs between the two groups, unequal under-registration is not likely to affect the outcome of the study. Under-registration or differences in definition of certain complications might also explain the differences found in non-fatal strokes, deaths, nerve injuries, and bleeding. Second, the results may have been confounded by the fact that the two strategies were performed in different hospitals, although both the participating hospitals are very comparable midsize teaching hospitals using similar guidelines and standards. Third, both cohorts consist of relatively small numbers of patients, with even smaller numbers of adverse events. The sample size is too small to rule out a type II error for the stroke or death outcome between the SPM and EEG/TCD cohorts. Therefore, we can only make cautious statements regarding the true effect of both strategies in terms of stroke or death rate. The rates found in our series do, however, correspond with those found in the literature. Fourth, as discussed earlier, the cutoff point for shunt insertion in the SPM cohort was quite high. While excessive shunt use is beneficial for training purposes, it may increase the intraoperative stroke risk. We do not know whether more neurologic events would have occurred if a lower value had been used, which, in case, would affect the results of the cost-effectiveness analysis. Finally, this study only addresses SPM and EEG/TCD as decision-making modality. There are, however, many more strategies advocated. Inclusion of other modalities too would be of value when looking for the most cost-effective strategy.

In conclusion, although this study is limited by its small sample size and retrospective nature, the primary clinical outcomes found are comparable to those in the literature. There is, however, a significant difference in admission between both strategies. Therefore, SPM might, although nowadays virtually abandoned for reasons, still be considered as a modality to indicate the need for shunting from a cost-effectiveness point of view.
